# Household Transmission of *Vibrio cholerae* in Bangladesh

**DOI:** 10.1371/journal.pntd.0003314

**Published:** 2014-11-20

**Authors:** Jonathan D. Sugimoto, Amanda A. Koepke, Eben E. Kenah, M. Elizabeth Halloran, Fahima Chowdhury, Ashraful I. Khan, Regina C. LaRocque, Yang Yang, Edward T. Ryan, Firdausi Qadri, Stephen B. Calderwood, Jason B. Harris, Ira M. Longini

**Affiliations:** 1 Center for Statistics and Quantitative Infectious Diseases, Department of Biostatistics, University of Florida, Gainesville, Florida, United States of America; 2 Department of Epidemiology, University of Florida, Gainesville, Florida, United States of America; 3 Emerging Pathogens Institute, University of Florida, Gainesville, Florida, United States of America; 4 Center for Statistics and Quantitative Infectious Diseases, Vaccine and Infectious Disease Division, Fred Hutchinson Cancer Research Center, Seattle, Washington, United States of America; 5 Department of Statistics, University of Washington, Seattle, Washington, United States of America; 6 Department of Biostatistics, University of Florida, Gainesville, Florida, United States of America; 7 Department of Biostatistics, University of Washington, Seattle, Washington, United States of America; 8 Centre for Vaccine Sciences (CVS), International Centre for Diarrhoeal Disease Research, Bangladesh, Mohakhali Dhaka, Bangladesh; 9 Division of Infectious Diseases, Massachusetts General Hospital, Boston, Massachusetts, United States of America; 10 Department of Medicine, Harvard Medical School, Boston, Massachusetts, United States of America; 11 Department of Immunology and Infectious Diseases, Harvard School of Public Health, Boston, Massachusetts, United States of America; 12 Department of Microbiology and Immunobiology, Harvard Medical School, Boston, Massachusetts, United States of America; 13 Department of Pediatrics, Harvard Medical School, Boston, Massachusetts, United States of America; University of California, San Diego, School of Medicine, United States of America

## Abstract

**Background:**

*Vibrio cholerae* infections cluster in households. This study's objective was to quantify the relative contribution of direct, within-household exposure (for example, via contamination of household food, water, or surfaces) to endemic cholera transmission. Quantifying the relative contribution of direct exposure is important for planning effective prevention and control measures.

**Methodology/Principal Findings:**

Symptom histories and multiple blood and fecal specimens were prospectively collected from household members of hospital-ascertained cholera cases in Bangladesh from 2001–2006. We estimated the probabilities of cholera transmission through 1) direct exposure within the household and 2) contact with community-based sources of infection. The natural history of cholera infection and covariate effects on transmission were considered. Significant direct transmission (p-value<0.0001) occurred among 1414 members of 364 households. Fecal shedding of O1 El Tor Ogawa was associated with a 4.9% (95% confidence interval: 0.9%–22.8%) risk of infection among household contacts through direct exposure during an 11-day infectious period (mean length). The estimated 11-day risk of O1 El Tor Ogawa infection through exposure to community-based sources was 2.5% (0.8%–8.0%). The corresponding estimated risks for O1 El Tor Inaba and O139 infection were 3.7% (0.7%–16.6%) and 8.2% (2.1%–27.1%) through direct exposure, and 3.4% (1.7%–6.7%) and 2.0% (0.5%–7.3%) through community-based exposure. Children under 5 years-old were at elevated risk of infection. Limitations of the study may have led to an underestimation of the true risk of cholera infection. For instance, available covariate data may have incompletely characterized levels of pre-existing immunity to cholera infection. Transmission via direct exposure occurring outside of the household was not considered.

**Conclusions:**

Direct exposure contributes substantially to endemic transmission of symptomatic cholera in an urban setting. We provide the first estimate of the transmissibility of endemic cholera within prospectively-followed members of households. The role of direct transmission must be considered when planning cholera control activities.

## Author Summary

Since John Snow's ground-breaking investigations of the devastating outbreaks in 19^th^-century London, cholera has been considered the quintessential waterborne human infection, transmitting via fecal contamination of environmental water sources. Recently, renewed interest has been paid to the potential importance of transmission through direct exposure within close-contact groups, such as, via fecal contamination of surfaces, food, or drinking water within households. Significant direct transmission of cholera within close contact groups would represent a new target for innovative prevention and control strategies. We estimated the probability of transmission 1) via direct contact within 364 urban households located in an endemic cholera setting (Dhaka, Bangladesh) and 2) via exposure to sources located outside of these households. In this setting we estimated a 4 to 8 percent probability of becoming infected with cholera via direct exposure within households in this setting versus a 2 to 3 percent likelihood of infection due to exposure to external sources over a comparable time period. Our results demonstrate that direct (within-household) transmission is a significant component of endemic cholera transmission, suggesting that biomedical and behavioral-modification interventions specifically targeting this mode of transmission could substantially reduce the cholera burden in this type of setting.

## Introduction

Cholera disproportionately affects less-developed areas of Asia, Africa, and Latin America, leading to an estimated 3–5 million cases and 100–130 thousand deaths per year [1]. *Vibrio cholerae* O1/O139 transmission is associated with two general modes of exposure to infection. Community-to-person transmission results from ingestion of contaminated water from environmental sources [2]. Direct transmission results from exposure to food, water, and surfaces shared by a cluster of individuals, such as a household, and contaminated by an infectious member of the cluster [1].

The relative contributions of community-to-person and direct exposure to endemic cholera transmission are subject to ongoing debate [2]–[7]. Community-to-person exposure is a well-established mode of transmission for cholera infection [8]–[11], whereas the relative contribution of direct exposure is poorly quantified. Evidence of herd immunity from cholera vaccine studies [12]–[14], reports from cholera outbreak investigations [15]–[17], the clustering of *V. cholerae* clones by household [18], and mathematical modeling of epidemics [3]–[5] suggest that direct transmission is an important component of overall transmission. John Snow himself believed that cholera transmission had a direct as well as a waterborne component. After describing many case studies of potential cholera transmission in England during the mid 1800's, Snow stated: “Besides the facts above mentioned, which prove that cholera is communicated from person to person, …” [19].

The effectiveness of interventions for the control of endemic cholera depends, in part, upon the prevailing mode of transmission. Dominant transmission through direct exposure is expected to generate clusters of cases within close contact groups, such as households. Transmission primarily via community-to-person exposure is expected to generate a more diffuse spatial pattern of cases within a population. Knowledge of the dominant mode of transmission is important for informing the selection of optimal intervention strategies, such as vaccination strategies for preventing ongoing cholera transmission in Haiti [20].

Here, we estimate the probabilities of endemic *V. cholerae* O1/O139 transmission through 1) exposure to community sources of infection and 2) direct exposure within the household. The modifying role of covariates and aspects of the natural history of *V. cholerae* infection are also described. To our knowledge, this study provides the first estimates of the transmission potential of endemic cholera via direct exposure among the members of prospectively-followed households.

## Materials and Methods

### Ethics statement

The Ethical and Research Review Committees of the International Centre for Diarrhoeal Disease Research in Dhaka, Bangladesh (icddr,b) and the Institutional Review Board of the Massachusetts General Hospital reviewed and approved this study. Written informed consent was collected from all participants, with consent provided by a parent or legal guardian for participants under 18 years-old. This analysis was conducted using de-identified data.

### Study design

A case-ascertained [21] study was conducted from January 2001 to May 2006 among households of Dhaka, Bangladesh, as previously described [18], [22]–[24]. Individuals older than six months of age presenting to the hospital of the icddr,b with severe acute watery diarrhea, stool positive for *V. cholerae* infection by culture, and without a history of significant co-morbidities were selected for inclusion as index cases. Antimicrobial therapy was provided to all index cases, as per the standard clinical practice for the management of acute watery diarrhea at the icddr,b. The study timeline is expressed relative to the enrollment day of the index case (day 1). Written informed consent was requested from the members of the index case's household on day 2 of the study. Households are defined as individuals who ate from the same cooking pot during the preceding three days. For children under 18 years of age, informed consent was requested from a parent or legal guardian. Consenting household members were enrolled if they were not participating in other icddr,b studies.

### Data collection

**Figure 1:**
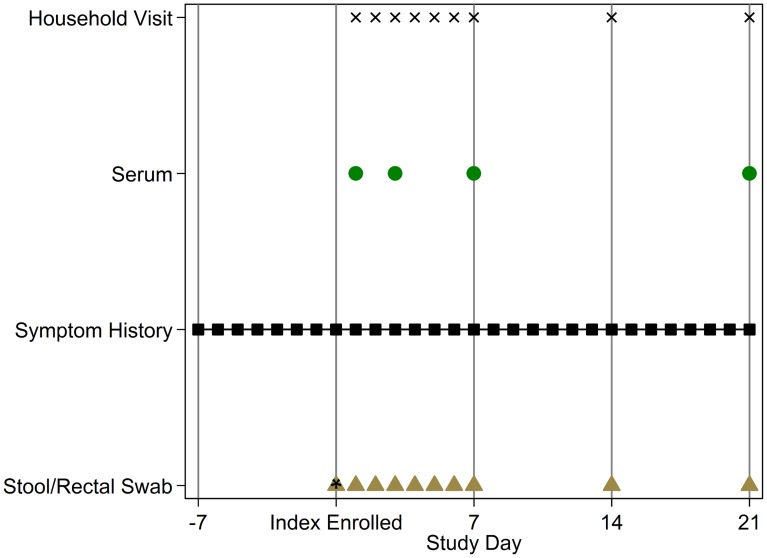
Survey data and specimen collection schedule for each study household, relative to the enrollment date of the household's index cholera infection (study day 1). The “*” denotes the day on which stool/rectal swab specimens were only collected from the index cholera infections.

Figure 1 describes the data collection schedule for each household. Upon enrollment, stool samples were obtained from the index cases. Study staff visited each household on days 2 through 7, 14, and 21. On day 2, information about age and sex was collected for all enrolled household members, along with an eight-day clinical history for preceding symptoms of diarrheal disease, *i.e.*, for days −7 to 1 (there was no day 0). Similar seven-day clinical histories were collected on days 7, 14, and 21.

Rectal swabs were collected from all study participants on days 2 through 7, 14, and 21. Blood specimens were collected from all study participants on days 2, 4, 7, and 21. Using previously-described methods [22], stool samples and rectal swabs were tested for *V. cholerae*, and blood specimens were assayed for vibriocidal antibody titers and ABO blood type. The serogroup (O1 or O139) and serotype of serogroup O1 El Tor biotype (Ogawa or Inaba) were determined for positive stool specimens.

### Statistical analysis

#### Outcome case definitions

Cholera infection by serogroup-serotype was the primary outcome for this analysis. Evidence of *V. cholerae* infection was defined as the occurrence of one or both of the following: a positive stool/rectal swab culture for *V. cholerae* O1/O139 or a ≥4-fold rise in serum vibriocidal antibody titer. A symptomatic cholera case, a secondary outcome, was defined as demonstrating evidence of *V. cholerae* infection plus the presence of watery diarrhea (≥3 watery stools per day) during the study period. Onset of symptoms was defined as the first appearance of watery diarrhea.

#### Latent and infectious periods

For the purposes of our analysis, only individuals with evidence of *V. cholerae* infection and at least one positive stool/rectal swab specimen for *V. cholerae* O1/O139 were considered infectious. The nature of the stool/rectal swab collection schedule (Figure 1) led to the onset day for infectiousness (defined as the first day that a specimen was or would have been observed to be positive had collection occurred daily during the study period) being unobserved, *i.e.*, left or interval censored, for some infectious individuals. Our analysis accounted for these censored observations.

The length of the latent period was assumed to follow a uniform distribution from 1 to 5 (mean of 3) days [2], [25]. The length of the infectious period was assumed to last from 1 to 14 days [14]. Among non-index case household members for whom the onset of shedding was observed (*i.e.*, a stool/rectal swab specimen collected on an earlier study day tested negative for cholera), we plot the daily probability of shedding after the onset of infectiousness (Figure S1 in Text S1). The probability density function for the infectious period was defined by fitting a non-parametric Loess smoothing kernel (bandwidth of 0.4) to the data in Figure S1 in Text S1.

All household members whose onset day for infectiousness was on or before that of the index case were classified as primary cases. Every other member was defined as a household contact. Household contacts included non-primary cholera infections and individuals without evidence of infection by the end of observation.

#### Covariates

We considered several known or suspected predictors of cholera risk. The continuous covariate age was categorized into three epidemiologically relevant risk strata [1], [2]: 0–4 years, 5–17 years, and 18 and older. Because O blood group has been associated with an increased risk for severe symptomatic cholera [26], [27], ABO blood group was classified as either O or non-O. Initial serum vibriocidal antibody titer was considered a measure of pre-existing immunity and defined as the measurement for day 2 (day 4 for the one individual missing a measurement for day 2). Households were excluded from the analysis if any enrolled member was missing all vibriocidal antibody titer measurements. Student t-tests (type I error rate of 0.05) were used to compare the equality of the distributions of covariates and the probability of *V. cholerae* detection in stool/rectal swab specimens between groups (for example, among the members of the excluded versus the included households).

#### Transmission model

**Figure 2:**
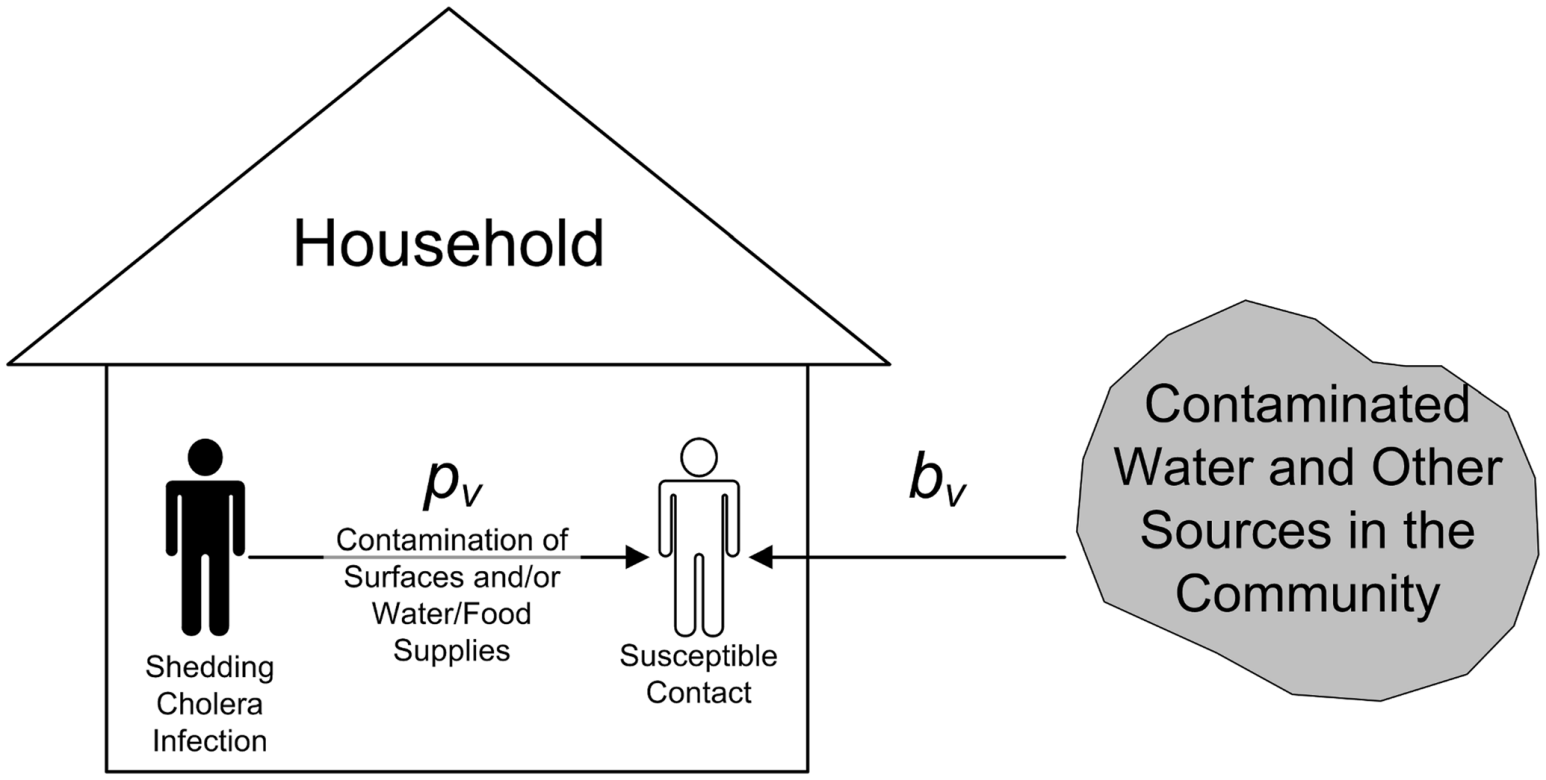
Schematic illustration of the transmission model. The probability 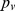 represents the daily risk that a susceptible contact (hollow figure) will subsequently develop cholera infection by serogroup-serotype *v* after exposure to household surfaces or water/food supplies contaminated by a member infected with and shedding *v* (black figure). The probability 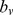 represents the susceptible contact's daily risk of cholera infection resulting from exposure to sources of infection located outside of his/her household. The corresponding epidemiologic summary measures for 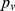 and 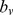 are the household secondary attack rate, or 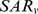, and the community probability of infection, or 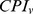 (see the text for parameter definitions).

We consider two modes of serogroup-serotype specific exposure to cholera infection (Figure 2): either through direct contact within the household or through community-to-person contact outside of the home. It is assumed that all members of a household come into daily contact both with each other and with potentially-contaminated sources of infection in the community. The parameters of interest are the serogroup-serotype specific probabilities of transmission per daily direct contact (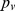) and infection per daily community-to-person contact (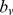), where 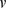 equals ‘Ogawa’ for O1 El Tor Ogawa, ‘Inaba’ for O1 El Tor Inaba, and ‘O139’ for O139 infection.

The direct transmission and community-to-person infection probabilities were estimated using a previously-described statistical model [21] (see Text S1). Similar models have been used to estimate transmission parameters and/or intervention effects for influenza, dengue, meningococcal, and pneumococcal infection [28]. To account for the potential selection bias associated with enrolling only households containing at least one symptomatic cholera case, this model considered the exposure of household contacts to primary cases, but did not include the latter individuals as incident cases in the estimation of 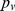 and 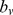. This model also adjusts for right censoring in the observed onset times for infectiousness. The null hypothesis of no evidence for direct transmission, *i.e.*, 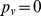, was tested using a likelihood ratio test [29] at a type I error rate of 0.05.

Extending the basic transmission model, we estimated the effects of covariates on susceptibility to cholera infection. Univariate models were fit for the effects of age group, sex, O blood group, and initial serum vibriocidal titer on susceptibility to infection. A multivariate model co-estimated the effects of all of these covariates. Because the sensitivity and specificity of serum vibriocidal antibody titer as a measure of pre-existing immunity to cholera infection may differ by serogroup-serotype [30], models assessing the effects of this covariate incorporated two additional terms for the multiplicative interaction between vibriocidal titer and serogroup-serotype.

The household secondary attack rate (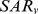) and the community probability of infection (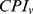) are the respective epidemiologic summary measures for 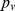 and 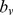. Following Yang *et al.* [21], we defined the 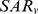 as the average proportion of household contacts infected by an individual during his/her infectious period, or 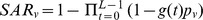, where 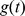 is the probability that a cholera infection remained infective on day 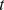 of an infectious period with maximum length 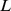. For an observation period of length 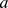, the 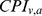 was defined as the cumulative risk of infection from a source located outside of the household, or 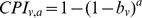. We consider a value of 14 days for 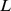 and values of 11 (corresponds to the mean length of an infectious period with 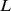 = 14) and 30 (one month) days for 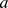.

A previously-reported expectation-maximization (EM) algorithm for this transmission model [31] was used to account for two quantities that were unobserved for some infected participants: the onset day for infectiousness and the serogroup-serotype of the infecting vibrios. For left-censored observations for the onset of infectiousness, the EM algorithm iterated over a period of time immediately prior to the enrollment date of the household index case. This period of time was defined as the maximum length of the infectious period (14 days) minus the number of days after the study enrollment during which *V. cholerae* continued to be detected in a stool/rectal swab specimen. Serogroup-serotype was unobserved for infections detected solely through a rise in serum vibriocidal antibody titers (Table S1 in Text S1).

#### Assessing model fit

We assessed the fit to the observed data for both the unadjusted basic transmission model (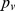-and-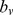) and an unadjusted 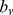-only transmission model (*e.g.*, the latter model only allowed for infection due to community-to-person exposure). The fit was assessed by comparing the observed final size distribution for infection to the empirically-approximated expected distribution generated through a parametric bootstrapping procedure [32]. The final size distribution is defined as the frequency of study households by the number of enrolled members and the number of infections among these members by the end of study follow-up. The expected final size distribution for each model was approximated empirically by simulating 1500 epidemics (each 34 days long) within a synthetic population whose structure was identical to the study population. Simulations used values for the serogroup-serotype specific transmission parameters drawn from a multivariate distribution defined by the estimates from the data and the corresponding variance-covariance matrix. Because the serogroup-serotype information was missing for a proportion of the observed infections, assessment of model fit was only conducted for the final size distribution for infections of all types. A chi-squared test was performed for the null hypothesis of no difference (type I error rate of 0.05) between the observed final size distribution and the mean of the empirical expected distribution.

The left-censoring of the onset of infectiousness for index and co-primary infections in the observed data necessitated the weighted selection of the corresponding onset dates used for each simulated epidemic. For each household, we enumerated every possible combination of dates for the onset of infectiousness among the index and co-primary infections. For each simulated epidemic, the selection of one of these possible combinations of onset dates was weighted by its expected likelihood during the final iteration of the EM algorithm used to fit the model to the data.

#### Software applications

Inference with the transmission models and the related parametric bootstrapping procedures were both conducted using TranStat, a publically-available software application (http://csquid.org/software/transtat/). Microsoft Visio 2010 (Microsoft Corp., Redmond, Washington State) was used to generate Figure 2. All other aspects of the analysis, including the chi-squared test for the model fit (*tabulate* command), were conducted using STATA version 12 (Stata Corp. LP, College Station, Texas).

## Results

### Study population

The study enrolled 1491 individuals living in 399 households (one index case each). We excluded 24 households with only one enrolled member (the index case) from the analysis, because they impart no information about transmission. An additional 11 households (53 members) were excluded from the analysis for lack of serum vibriocidal antibody titer measurements. The resulting study population of 1414 people lived in 364 households. Table S1 in Text S1 provides the frequency of the households included in the analysis by the number of enrolled members, infections among the enrolled, and the serogroup-serotype of infection. The 53 enrolled study members who were excluded from the analysis because of missing vibriocidal antibody titer data did not substantially differ from the study population in the distributions of age (p<0.580), sex (p<0.981), the prevalence of O blood group (p<0.320), the attack rate for watery diarrhea (p<0.508), or the proportion of members with at least one stool/rectal swab specimen positive for *V. cholerae* (p<0.098) (Table 1).

**Table 1:** Descriptive statistics for the study population.

Statistic	Excluded Households^a^ (N = 11)		Included Households (N = 364)							
			All Members		Index Cholera Infections		Household Contacts			
							Non-Index Cholera Infections		Uninfected Contacts	
**Number of individuals** (% of All Members)	53		1414		364	(25.7)	318	(22.5)	732	(51.8)
**Individuals per household**	4.8	(1.4)	3.9	(1.5)	1.0	(0.0)	0.9	(1.1)	2.0	(1.4)
Mean (SD)										
**Baseline characteristics**										
*Age* (years)										
Mean (SD)	23.2	(17.0)	22.0	(15.0)	23.8	(14.3)	18.8	(14.8)	22.5	(15.2)
Median	17.9		20.0		22.6		15.0		19.8	
*Age group*: N (%)										
0–4 years	4	(7.5)	167	(11.8)	23	(6.3)	64	(20.1)	80	(10.9)
5–17 years	23	(43.4)	477	(33.7)	112	(30.8)	109	(34.3)	256	(35.0)
≥18 years	26	(49.1)	770	(54.5)	229	(62.9)	145	(45.6)	396	(54.1)
*Male sex*: N (%)	26	(49.1)	696	(49.2)	161	(44.2)	163	(51.3)	372	(50.8)
*O blood group*: N (%)	15	(28.3)	494	(34.9)	150	(41.2)	94	(29.6)	250	(34.2)
*Initial serum vibriocidal antibody titer* b										
Mean (SD)	1.8	(0.8)	1.8	(0.9)	1.8	(0.8)	1.7	(0.9)	2.0	(0.9)
Median	1.9		1.9		1.9		1.6		1.9	
**Watery diarrhea**: N (%)	26	(49.1)	759	(53.7)	364	(100.0)	180	(56.7)	215	(29.4)
**Evidence of infection**										
*a) Positive rectal swab or stool specimen*: N (%)	16	(30.2)	588	(41.6)	364	(100.0)	224	(70.4)	NA	
O1 El Tor Ogawa: N (%)	8	(15.1)	192	(13.6)	123	(33.8)	64	(21.7)	NA	
O1 El Tor Inaba: N (%)	7	(13.2)	278	(19.7)	180	(49.5)	98	(30.8)	NA	
O139: N (%)	1	(1.9)	118	(8.3)	61	(16.8)	57	(17.9)	NA	
*b) 4-fold rise in vibriocidal antibody titer*: N (%)	NA		568	(40.2)	342	(94.0)	226	(71.1)	NA	
*a) AND b)*: N (%)	NA		474	(33.5)	342	(94.0)	132	(41.5)	NA	
*a) OR b)*: N (%)	NA		682	(48.2)	364	(100.0)	318	(100.0)	NA	

*Footnotes*

N (%), Number of individuals (percentage of the column total in the first row); NA, Not applicable; SD, Standard deviation.

a Excluded because one or more members did not have vibriocidal antibody titer measurements. This does not include the 24 households that were excluded for having only one member.

b Defined as the base-ten logarithm of the vibriocidal antibody titer of the initial serum specimen collected on day 2 of the household observation period. For one individual, the serum specimen collected on day 4 was used due to a missing measurement for day 2.

Non-primary cholera infections were significantly younger than index cholera infections (p<0.001), but not uninfected household contacts (p<0.200) (Table 1). The sex ratio and the prevalence of O blood group were similar for infected and uninfected household contacts (p<0.896 and p<0.145, respectively). When compared to household contacts, index cases were more commonly female (p<0.027) and of the O blood group (p<0.004).

**Figure 3:**
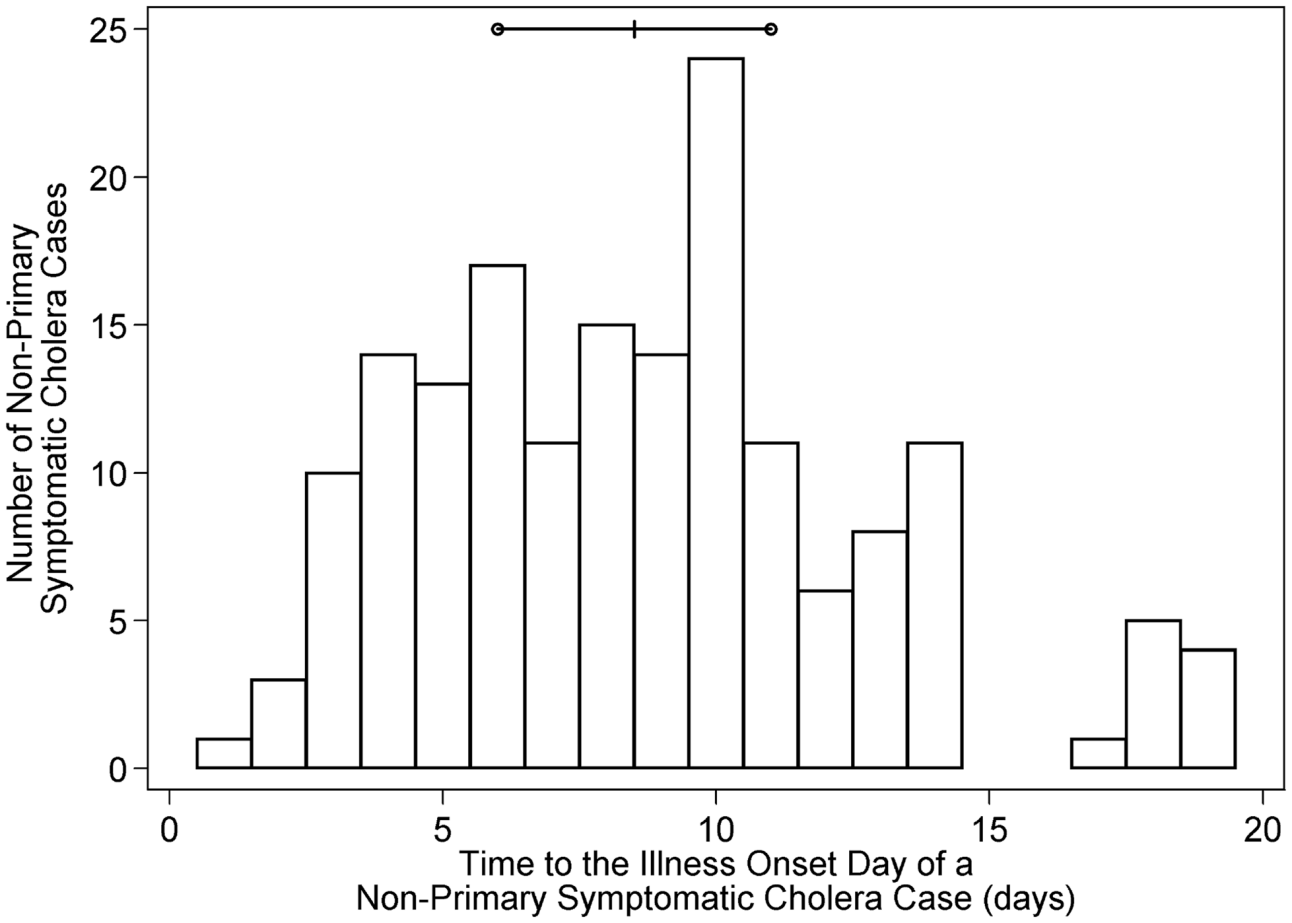
The number of days between the illness onset dates of household primary and non-primary symptomatic *Vibrio cholerae* cases (all serogroup-serotypes). Primary symptomatic cholera cases are defined as enrolled household members meeting the case definition for a symptomatic cholera case and whose symptom onset date was on or before that of the household's index infection. All other symptomatic cholera cases are classified as non-primary. The horizontal line represents the interquartile range (25^th^ through the 75^th^ percentile), with the median denoted by the vertical crossbar.

Among the 1050 household contacts, 22.5% (318) developed *V. cholerae* infection (Table 1). The proportion of non-index *V. cholerae* infections with watery diarrhea was 56.7% (180 of 318). The number of days between the symptom onset dates of the primary (onset of symptoms on or before onset in the household index infection) and non-primary symptomatic cholera cases in a household was not uniformly distributed (Figure 3); the mean (standard deviation) duration was 9 (4) days.

### Transmission

For each serogroup-serotype, we rejected the null hypothesis of no direct transmission (p-value<0.001). The SAR estimates were 4.9% (95% CI: 0.9%–22.8%) for 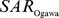, 3.7% (95% CI: 0.7%–16.6%) for 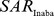, and 8.2% (95% CI: 2.1%–27.1%) for 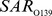. The CPI estimates for a comparable 11-day period were 2.5% (0.8%–8.0%) for 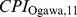, 3.4% (1.7%–6.7%) for 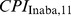, and 2.0% (0.5%–7.3%) for 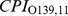. Serogroup-serotype specific estimates for the risk of infection resulting from a month (30 days) of community-to-person exposure were 6.7% (2.1%–20.4%) for 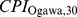, 9.0% (4.5%–17.3%) for 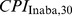, and 5.3% (1.4%–18.7%) for 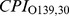. For all serogroup-serotypes combined, the estimated risk of cholera infection associated with community-to-person exposure varied by calendar month of the year (Figure S2).

### Covariate effects

In the univariate and multivariate transmission models, children under five years of age were significantly more susceptible than adults 18 years and older to cholera infection (Table 2). The susceptibility of children 5–17 years-old to cholera infection did not significantly differ from that of adults 18 years and older. There was no evidence of significant differences in the susceptibility of contacts based upon either sex or ABO blood group.

**Table 2:** Covariate effects estimated by the univariate and multivariate transmission models.

Covariate	Odds Ratio (95% Confidence Interval)			
	Univariate Models		Multivariate Model	
**Age group** (years)				
0–4 versus ≥18	1.66	(1.17–2.36)	1.54	(1.10–2.15)
5–17 versus ≥18	0.95	(0.70–1.28)	0.92	(0.68–1.24)
**Sex**				
Male versus Female	1.01	(0.78–1.32)	0.98	(0.75–1.27)
**ABO blood group**				
O versus Non-O	0.91	(0.68–1.22)	0.93	(0.71–1.22)
**Initial vibriocidal antibody titer, by serogroup-serotype** (per 2-fold greater titer)				
O1 El Tor Ogawa	0.90	(0.76–1.05)	0.91	(0.83–0.99)
O1 El Tor Inaba	1.01	(0.91–1.11)	1.01	(0.92–1.10)
O139	0.82	(0.55–1.22)	0.83	(0.64–1.08)

Serum vibriocidal antibody titers demonstrated some protection against infection by O1 El Tor Ogawa and O139. This protection was only statistically significant for O1 El Tor Ogawa in the multivariate model, providing an estimated 9% (95% CI: 1%–17%) reduction in susceptibility to infection per two-fold higher initial titer for serum vibriocidal antibody. The magnitude of protection against O139 infection was larger than the effect for O1 El Tor Ogawa, but lacked statistical significance in every model.

### Model fit

The unadjusted transmission model, both including (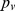-and-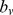) and excluding (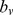-only) transmission through direct exposure, demonstrated adequate fit to the study data (Figure S3 in Text S1). For both unadjusted transmission models, chi-squared tests rejected the null hypothesis for a difference between the observed and expected final size distributions (p-value< = 0.001). There was little evidence to suggest that the quality of the fit of the unadjusted transmission model including direct transmission differed from that of the model excluding this mode of exposure.

## Discussion

To the best knowledge of our knowledge, this is the first report quantifying the transmissibility of *V. cholerae* O1/O139 infection in households through direct contact using prospectively-observed individual-level data. We estimate that cholera infections with evidence of fecal shedding of vibrios, on average, infected 4% to 8% of susceptible household contacts over the course of an 11-day infectious period (mean length). Our results demonstrate that direct exposure to infectious household members plays an important role in the transmission of *V. cholerae* O1/O139 in a population where cholera has been historically endemic.

This observation is consistent with mathematical and epidemic modeling studies [4], [5], as well as the results of epidemiologic [12]–[17] and environmental contamination [33] studies. In urban Kolkata, India, (a setting similar to Dhaka, Bangladesh), the level of fecal contamination of water sources was assessed in a random set of households recently reporting a case of diarrhea [33]. Intriguingly, seven percent (7%) of samples collected by this study from water stored within the home were reported to harbor *V. cholerae* [33], which is strikingly similar in value to our estimates for the household secondary attack rate, 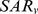.

Our results support the conclusion that during an outbreak of cholera in a household in an endemic zone, the risk of infection due to exposure to non-household sources is approximately 2 to 4 times lower in magnitude than the risk attributable to direct exposure. The nature of community-based sources of cholera infection, *i.e.*, through contaminated water sources, suggests that the duration of exposure to sources of this type is much longer than the duration of direct exposure to an infectious household member. Indeed, our results estimate that the risk of infection due to exposure to community-based sources of infection is approximately 5 to 9 percent for a 30-day period, and the level of risk of infection from community-based exposure varies some throughout the calendar year, but is consistently greater than 0% (Figure S2 in Text S1).

Taken together, the results of our study argue for an epidemiologic model for endemic cholera transmission in an urban setting where there is a persistent low-level of exposure to community-based sources of *V. cholerae* infection. Once an individual is infected through exposure to a community-based source of infection, the risk profile of this individual's close contacts (*i.e.*, household members) changes substantially to a scenario where the greatest risk of infection is through direct transmission. Both the small number of individuals residing within a household and the relatively short duration of infectiousness would be expected to limit the duration of direct transmission within the household. Once the level of direct transmission subsides, then the risk profile for infection would revert back to the initial state. Of note, we cannot comment on the actual mechanism of possible direct transmission within the household. Considering the relatively high infectious dose required and usual mode of transmission of *V. cholerae*, direct transmission within the household is likely to have involved contamination of a shared water source or storage container or preparation and/or storage of food by the shedding index case.

### Limitations

This study and analysis have several limitations that are expected to result in underestimation of the true serogroup-serotype specific household secondary attack rates for *V. cholerae* infection. Initial serum vibriocidal antibody titers may incompletely account for levels of pre-existing immunity to *V. cholerae* infection [30]. In addition, we only considered direct transmission within the households of study participants. Some of the transmission attributed to community-to-person exposure may have actually resulted from direct exposure outside of the participant's own household.

Theoretically, the observed pattern of cholera infections could have been solely attributable to community-to-person transmission. The inability to discern between transmission of *V. cholerae* through exposure to direct versus short-duration community-based sources of infection has been demonstrated through mathematical modeling investigations using mass action transmission models [3], [4]. If the only source of exposure to cholera infections in one of our study households was a common community-based source of infection that occurred on a single day, a pattern of illness onset dates consistent with acute direct transmission could occur. If this scenario occurred in every household enrolled in this study, our statistical model would errantly attribute some or all transmission to direct exposure. If a community-based source of infection exposed the members of a household for a period longer than one day (a plausible scenario), our statistical model would be able to differentiate between transmission due to that source and that resulting from direct exposure within the household. The fact that the mean time between the onset of symptoms in primary and associated non-primary symptomatic cholera cases was longer than the maximum length of the incubation period supports the assertion that direct and/or multi-day community-based sources of exposure to infection were operating in this population during this study.

The current study categorizes the cholera infections by bacterial phenotype, *i.e.*, biotype, serogroup, and serotype. The primary limitation of using this categorization schema relates to the substantial genetic variation evident among vibrios of the same bacterial phenotype. Since the probability of direct transmission of cholera within households is likely to be strongly associated with genetic similarity of the infecting vibrios, this analysis would certainly have been enhanced by incorporation of measures of the genetic similarity between the cholera bacteria isolated from the participants in the same household. If this type of information had been available for the current study, our analysis could have either 1) estimated genotype specific SAR's (analogous to the approach used here to estimate SAR's by bacterial phenotype) or 2) directly incorporated measures of genetic distance into the likelihood for the transmission model. Whole genome sequencing has been used characterize genetic variability among clinical *V. cholerae* isolates, for example, to characterize the origins of the recent outbreak of cholera in Haiti [34]. Ongoing work by the authors [35] seeks to address this limitation through a combination of ongoing field studies and statistical methodologic research.

### Conclusions

Cholera remains an important public health issue for low-resource settings with limited public health facilities. Recent experiences in Zimbabwe and Haiti underscore the urgent need for effective intervention strategies [1], [36]. Our results demonstrate that exposure through direct contact significantly contributes to the endemic transmission of cholera infection. Greater emphasis needs to be placed on implementing interventions targeting transmission through direct exposure. Consideration should be given to evaluating the utility of pre-emptive administration of antimicrobial agents to the household contacts of patients with cholera in areas lacking adequate sanitation. Vaccination of entire households prior to the onset of seasonal transmission may allow for additional control of transmission by bolstering existing immunity among members, thereby reducing the level of direct transmission and protecting against community-to-person transmission from other contaminated sources.

## Supporting Information

Checklist S1 STROBE checklist.
(DOCX)

Text S1 Supplementary information.
(DOCX)
