# Supplementary material for: Household Transmission of Vibrio cholerae in Bangladesh
Source: PLoS Negl Trop Dis. 2014 Nov 20;8(11):e3314. doi: 10.1371/journal.pntd.0003314 (PMC4238997; doi:10.1371/journal.pntd.0003314)
Supplement: Text S1 — Supplementary information. (DOCX) [file pntd.0003314.s002.docx]

**Household Transmission of *Vibrio cholerae* in Bangladesh**

**TEXT S1**

*Authors*

Jonathan D. Sugimoto ^a,b,c,d^

Amanda A. Koepke ^a,d,e^

Eben E. Kenah ^a,c,f^

M. Elizabeth Halloran ^a,d,g^

Fahima Chowdhury ^h^

Ashraful I. Khan ^h^

Regina C. LaRocque ^i,j^

Yang Yang ^a,c,f^

Edward T. Ryan ^i,j,k^

Firdausi Qadri ^h^

Stephen B. Calderwood ^i,j,l^

Jason B. Harris ^i,m^

Ira M. Longini, Jr. ^a,c,f^

*Affiliations*

^a^ Center for Statistics and Quantitative Infectious Diseases, Department of Biostatistics, University of Florida, P.O. Box 117450, Gainesville, FL 32610 USA

^b^ Department of Epidemiology, University of Florida, Gainesville, FL 32610 USA

^c^ Emerging Pathogens Institute, University of Florida, P.O. Box 100009, Gainesville, FL 32610 USA

^d^ Center for Statistics and Quantitative Infectious Diseases, Vaccine and Infectious Disease Division, Fred Hutchinson Cancer Research Center, 1100 Fairview Ave N, M2-C200, Seattle, WA 98109 USA

^e^ Department of Statistics, University of Washington, Box 354322, Seattle, WA 98195 USA

^f^ Department of Biostatistics, University of Florida, P.O. Box 117450, Gainesville, FL 32610 USA

^g^ Department of Biostatistics, University of Washington, Box 357232, Seattle, WA 98195 USA

^h^ Centre for Vaccine Sciences (CVS), International Centre for Diarrhoeal Disease Research, Bangladesh (icddr,b), 68, Shaheed Tajuddin Ahmed Sarani, Mohakhali Dhaka 1212, Bangladesh

^i^ Division of Infectious Diseases, Massachusetts General Hospital, 55 Fruit Street, Boston, MA 02114 USA

^j^ Department of Medicine, Harvard Medical School, 25 Shattuck Street, Boston, MA 02115 USA

^k^ Department of Immunology and Infectious Diseases, Harvard School of Public Health, 677 Huntington Avenue, Boston, MA 02115 USA

^l^ Department of Microbiology and Immunobiology, Harvard Medical School, 25 Shattuck Street, Boston, MA 02115 USA

^m^ Department of Pediatrics, Harvard Medical School, 25 Shattuck Street, Boston, MA 02115 USA

*Corresponding Author*

Ira M. Longini, Jr.

Center Statistics and Quantitative Infectious Diseases (CSQUID)

Department of Biostatistics

University of Florida

P.O. Box 117450

228 Buckman Drive, 4th floor Dauer Hall

Gainesville, FL 32610

Phone: 352-294-1937

## S1.1 Statistical transmission model

An extension [[1](#_ENREF_1)] of a chain-binomial model [[2](#_ENREF_2)] for the transmission of infectious diseases in close contact clusters implements a data augmentation approach, referred to as a hybrid expectation maximization (EM) and Monte Carlo EM (EM-MCEM) algorithm, to iterate over unobserved instances of the following quantities: an individual’s outcome status by the end of follow-up and/or the timing for the onset of infection. This algorithm is ‘hybrid’ in the sense that the MCEM is used to augment data for independent clusters of individuals to which the classical EM algorithm would be numerically infeasible to apply. This novel method is illustrated through the analysis [[1](#_ENREF_1)] of influenza transmission within Seattle households [[3](#_ENREF_3),[4](#_ENREF_4)].

The current work adapts this EM-MCEM algorithm [[1](#_ENREF_1)] to the analysis of case-ascertained study data describing the transmission of three distinct strains (serogroup-serotype combinations: O1 El Tor Ogawa, O1 El Tor Inaba, and O139) of *Vibrio cholerae* within urban households in Bangladesh. Though infection by each strain tends to cluster by household, there are still some households where members were infected with different strains or for which the infecting strain is unobserved for at least one infected member (see Table S1 in Text S1). Due to the short follow-up period for each household (28 days) relative to the 17-day generation interval (mean duration of latent period of 3 days [[5](#_ENREF_5)], plus the maximum length of the infectious period of 14 days [[6](#_ENREF_6)]) for cholera, it is assumed that enrolled participants members were only able to be infected once during study observation. There is no evidence from the study data to support that any participants were infected by multiple strains during the follow-up period. Therefore, this analysis adapts the existing model [[1](#_ENREF_1)] by adding a competing hazards assumption. All strains were assumed to be competing for susceptible hosts up to the time point of infection by one strain. For this analysis, infected hosts were no longer considered to be at risk for infection.

First, we describe the basic likelihood (similar to [[2](#_ENREF_2)]) for the transmission model with three competing strains, assuming complete observation of outcome status by the end of study follow-up, the onset time for infectiousness, and the strain of infecting vibrios. Then, we provide a brief description of the EM-MCEM algorithm. We refer the reader to [[1](#_ENREF_1)] for a more detailed description of the EM-MCEM algorithm.

*Basic likelihood.* Denote the size of the population in the study households by $N$. Let $V$ denote the number strains of type $v$, with $v=1,2, \text{and }3$. Let $H$ be the number of independent households, and let $d_{s}$ denote the index/primary case for household $s$, $s=0,1,\cdots,H$. Let $\tilde{t}_{i}$ be the day of onset of infectiousness (first evidence of *V. cholerae* in the stool specimen or rectal swab) for an infection of individual $i$, with the default $\tilde{t}_{i}=\infty$ if $i$ is not infected, $i=1,\cdots,N.$ Household members with onset of infectiousness on or before $\tilde{t}_{d_{s}}$ are considered co-primary cases. All household members who are not classified as primary or co-primary cases are considered household contacts.

We analyze the data as independent outbreaks in each of the $H$ household clusters. We estimate the probability $p_{v}$ of transmission of strain $v$ per daily within-household contact between members. In addition, each household contact may be exposed to infection via community-to-person contact with contaminated sources of water in the community or casual contact with other potential sources located outside of the household, leading to the daily probability $b_{v}$ of infection with strain $v$ via this transmission mode. A household member $j$ infected with strain $v$ is only considered infectious if there is evidence that s/he shed *V. cholerae* of that strain in his/her stool during the household outbreak.

Denote the last day of analysis for person $i$ as $T_{i}$, which is equal to day 28 for all individuals. The households in this analysis were sampled from the population using the case-ascertained study design [[2](#_ENREF_2)]. In this study design, each household $s$contains at least one index/primary case of cholera, leading to the potential for selection bias in the estimation of $p_{v}$ and $b_{v}$. As an adjustment for selection bias in studies with a case-ascertained design [[2](#_ENREF_2)], $T_{i}$ need not be defined for index/primary and co-primary cases, since their infection status does not contribute to the overall likelihood. However, the exposure of household contacts to infectious primary or co-primary cases is considered in the estimation of $p_{v}$.

We estimated the effects of covariates on $p_{v}$ and $b_{v}$. The $k$considered covariates are age-group [two binary indicator variables: $x_{1}$, 1 for children 0-4 years and 0 for all others, and $x_{2}$, 1 for children 5-17 years and 0 for all others]; gender [1 for males and 0 for females], denoted as $x_{3}$; ABO blood group [1 for O and 0 for non-O blood group], denoted as $x_{4}$; and vibriocidal serum antibody titer at the beginning of the household outbreak [a main effect, the base-2 logarithm, denoted as $x_{5}$, and the two terms, $x_{6}$ and $x_{7}$, for the multiplicative interaction between $x_{5}$ and strain $v$]. The effects of these covariates are estimated for susceptibility to infection. The probability, adjusted for all covariates, $P_{ijv}(t)$, that susceptible person $i$ was infected by strain $v$ via a contact with an infectious person $j$ on day $t$ is given by

$logit\left( P_{ijv}\left( t \right) \right)=logit\left( {g\left( t-\tilde{t_{j}} \right)}_{v}p_{v} \right)+\boldsymbol{\beta}^{\boldsymbol{\tau}}\boldsymbol{x}_{\boldsymbol{i}}$,

where ${g\left( t-\tilde{t_{j}} \right)}_{v}$ is the probability of $j$ being infectious on day given onset of infectiousness with strain $v$ on day $\tilde{t}_{j}$. ${g\left( t-\tilde{t_{j}} \right)}_{v}$ solely depends on $t-\tilde{t}_{j}$ and is assumed known. $\boldsymbol{x}_{\boldsymbol{i}}=\left( x_{i1},\ldots,x_{ik} \right)$ and $\boldsymbol{\beta}^{\boldsymbol{\tau}}=\left( \beta_{1},\ldots,\beta_{k} \right)^{\tau}$. $\beta x_{i}$ is equal to $\beta_{1}x_{1}+\beta_{2}x_{2}$, $\beta_{3}x_{3}$, $\beta_{4}x_{4}$, $\beta_{5}x_{5}+\beta_{6}x_{6}+\beta_{7}x_{7}$, and $\beta_{1}x_{1}+\beta_{2}x_{2}+\beta_{4}x_{4}+\beta_{5}x_{5}+\beta_{6}x_{6}+\beta_{7}x_{7}$ for the age-group, sex, ABO blood group, initial vibriocidal serum antibody titer, and multivariate (age-group, sex, ABO blood group, and initial vibriocidal serum antibody titer) adjusted models, respectively. The odds ratio for a covariate’s effect on susceptibility to infection is estimated as $e^{\boldsymbol{\beta}}$. Similarly, the covariate-adjusted probability, $B_{iv}(t)$, that a susceptible person $i$ is infected by strain $v$ via either contact with contaminated sources of water in the community or through a casual contact outside of the household on day $t$ is given by

$logit\left( B_{iv}\left( t \right) \right)=logit\left( b_{iv} \right)+\boldsymbol{\beta}^{\boldsymbol{\tau}}\boldsymbol{x}_{\boldsymbol{i}}$.

We assume that the infectious period has a maximum of duration of $\Delta$days. As a result, ${g\left( t-\tilde{t}_{j} \right)}_{v}>0$ for $\tilde{t}_{j}\leq t\leq\left( \tilde{t}_{j}+\Delta-1 \right)$ and is 0 otherwise. The probabilities ${g\left( t-\tilde{t}_{j} \right)}_{v}$ characterize the distribution of the infectious period for the disease (see Section S1.2 in Text S1 for a description of the empirical approximation of ${g\left( t-\tilde{t}_{j} \right)}_{v}$ employed for this analysis). We assume the same distribution ${g\left( t-\tilde{t}_{j} \right)}_{v}$ for all strains $v$.

Let $C_{i}^{b_{v}}(t)$ be the collection of community-to-person contacts and $C_{i}^{p_{v}}(t)$ be the collection of within-household contacts that person $i$ made with sources of exposure to strain $v$ on day $t$. Let stand for the empty set. The elements of $C_{i}^{p_{v}}$ are indexed by both the infectious person and type of within-household contact. For an individual $i$ infected with strain $l$ (same set of possible values as for $v$), $C_{i}^{p_{v}}\left( t\geq\tilde{t}_{i} \right)=\varphi$ and $C_{i}^{b_{v}}\left( t\geq\tilde{t}_{i} \right)=\varphi$ for all strains $v\neq l$. The last statement describes the competing hazards component of this multi-strain model. For all other times $t$, we have $C_{i}^{b_{v}}\left( t \right)=\{1\}$, and $C_{i}^{p_{v}}$ includes $(j)$ if a susceptible household member $i$ was exposed to a household member $j$ on day $t$ of infected individual $j$’s infectious period.

Let $I(\cdot)$ be the indicator function. The probability that a susceptible person $i$ escapes infection from all infectious sources on day $t$ is then given by

$$e_{i}\left( t \right)=\prod_{v=1}^{3} \left\{ \left\{ (1-B_{iv}\left( t \right)) \right\}^{1-I(C_{i}^{b_{v}}\left( t \right)=\varphi)}\left\{ \prod_{(j)\in C_{i}^{p_{v}}(t)} P_{ijv}(t) \right\}^{1-I(C_{i}^{p_{v}}\left( t \right)=\varphi)} \right\}$$

We additionally assume that the duration of the latent period has a known distribution, denoted by $\eta(\tilde{t}_{i}-t)$, *i.e.*, the probability of the onset of infectiousness on day $\tilde{t}_{i}$, given infection on day $t$. $\eta(\tilde{t}_{i}-t)$solely depends on $\tilde{t}_{i}-t$. Let $\varsigma_{min}$ and $\varsigma_{max}$be the minimum and maximum duration of the latent period, such that $\eta\left( \tilde{t}_{i}-t \right)>0$ only if $\tilde{t}_{i}-\varsigma_{max}\leq t\leq\tilde{t}_{i}-\varsigma_{min}$. Defining $\tilde{t}=\{\tilde{t}_{i}, i=1, \cdots, N\}$, we construct the likelihood for a household contact person $i$ as

$$L_{i}\left( \boldsymbol{b},\boldsymbol{p},\boldsymbol{\beta} | \tilde{t} \right)=\left[ \begin{matrix} \prod_{t=1}^{T_{i}} e_{i}(t) & i \text{is not infected} \\ \sum_{t=\tilde{t}_{i}-\varsigma_{max}}^{\tilde{t}_{i}-\varsigma_{min}} \left\{ \eta(\tilde{t}_{i}-t)(1-e_{i}\left( t \right))\prod_{\tau=t}^{t-1} e_{i}(\tau) \right\} & i \text{is infected} \end{matrix} \right.$$

, where $\boldsymbol{b=(}b_{1},b_{2},b_{3}\boldsymbol{)}$ and $\boldsymbol{p=(}p_{1},p_{2},p_{3}\boldsymbol{)}$ represent

To further adjust for selection bias in the case-ascertained design, the likelihood should be conditioned on the infectiousness status of person $i\in s$ on the day $\tilde{t}_{d_{s}}$. Following [[2](#_ENREF_2)], the marginal probability of having symptom onset later than $\tilde{t}_{d_{i}}$ is

$$L_{i}^{m}\left( \boldsymbol{b},\boldsymbol{p},\boldsymbol{\beta} | \tilde{t} \right)=\sum_{t=1}^{\tilde{t}_{d_{s}}-\varsigma_{min}} \left\{ \left( \prod_{\tau=1}^{t-1} e_{i}(\tau) \right)(1-e_{i}\left( t \right))\sum_{\tau>\tilde{t}_{d_{s}}} (\eta(\tau-t)) \right\}+\sum_{t=1}^{\tilde{t}_{d_{s}}-\varsigma_{min}} e_{i}(t)$$

where $\sum_{\tau>\tilde{t}_{d_{s}}} \left(\eta(\tau-t)\right)$ is the probability that the latent period is longer than $\tilde{t}_{d_{s}}-t$. Let $\Omega$ be the collection of people who are not primary cases. The joint conditional likelihood

$$L^{c}\left( \boldsymbol{b},\boldsymbol{p},\boldsymbol{\beta} | \tilde{t} \right)=\prod_{i\in\Omega} \frac{L_{i}\left( \boldsymbol{b},\boldsymbol{p},\boldsymbol{\beta} | \tilde{t} \right)}{L_{i}^{m}\left( \boldsymbol{b},\boldsymbol{p},\boldsymbol{\beta} | \tilde{t} \right)}$$

is maximized to obtain the maximum likelihood estimates (MLE).

To investigate the variation in the estimated community-to-person probability of infection throughout the calendar year, a variant of this transmission model was fit for infection by any serogroup-serotype, *i.e.*, $b_{x}$, where $x$ denotes all $v\in(1,2,3)$. This variant of the transmission model estimated a separate $b_{x}$ for community-to-person exposure occurring during each of the 12 calendar months of the year. A single parameter, $p_{x}$, representing transmission through direct exposure within the household was also included in this variant of the transmission model.

*Assumptions concerning the nature of missing information in the current dataset*. Since two different methods were used to assess every member of a household for signs of cholera infection during the study follow-up period (*i.e.*, monitoring stool/rectal swab specimens for vibrios and comparing vibriocidal antibody titers from serum samples collected at the beginning and later in the study follow-up period), household contacts that did not show any signs of infection by the end of the household outbreak are reasonably assumed to have either escaped infection, been pre-existing immune, or experience right-censoring of infection time by termination of study follow-up. Since this analysis adjusts for the effects of a proxy measure of pre-existing immunity to cholera infection (vibriocidal antibody titers measured from the serum specimens collected at the beginning of the household outbreak period), we assume that all pre-existing immunity to infection by strain $v$ was observed. Using previously specified methods [[2](#_ENREF_2)], this analysis accounted for potential right-censoring of the observed onset of infectiousness. Therefore, this analysis only iterated over the any unobserved values for the infecting serogroup-serotype ($v)$ and/or the onset time for infectiousness ($\tilde{t}_{j}$) using the hybrid EM-MCEM algorithm [[1](#_ENREF_1)].

*Brief summary of EM-MCEM algorithm*. The hybrid EM-MCEM algorithm for this transmission model [[1](#_ENREF_1)] relies on the assumption that households are independent clusters of individuals, *i.e.*, there is no interaction between members of the different households and membership is restricted to one household. Based upon the assumption of independence between households, imputation need only be done at the level of the household. Define $\delta_{h}$ as the number of possible realizations of the missing data for individuals in household $h$. For households whose members have completely observed data, $\delta_{h}=1$. Let $\boldsymbol{U}_{hk}^{\boldsymbol{*}}$ represent the collection of all possible realizations of the missing data. For this analysis, $\boldsymbol{U}_{hk}^{\boldsymbol{*}}$ is restricted to the set of possible realizations that did not violate the competing hazards assumption of this analysis.

The ‘hybrid’ nature of the EM-MCEM is evidenced by the following choice in the algorithm’s decision tree. If $\delta_{h}$ is too large, it will be numerically and computationally infeasible to use the EM algorithm. Therefore, an arbitrary cut-off value $J$ must be selected, where for values of $\delta_{h}>J$ the EM will be replaced by the MCEM algorithm (adapted from the algorithm proposed by [[7](#_ENREF_7)] for importance sampling). The basic EM-MCEM algorithm for estimating the parameter set $\psi=(b_{v},p_{v},\boldsymbol{\beta}|observed and unobserved data)$ is summarized from [[1](#_ENREF_1)] as follows:

1. Choose a value for $J$, and then households are partitioned into three groups: no data augmentation required ($\delta_{h}=1)$, data augmentation using the EM ($1>\delta_{h}\leq J$), and data augmentation using the MCEM ($\delta_{h}>J$).

2. Choose a value for $K$, the number of importance samples for the MCEM algorithm.

3. Choose a set $\psi^{(0)}$ of initial values for the parameters of the model. For households assigned to imputation by MCEM, use an MCMC algorithm to draw $K$ samples for the set of missing data among household members, $\boldsymbol{U}_{h}$, conditional upon the observed data and $\psi^{(0)}$. These samples will be represented by ${\hat{\boldsymbol{U}}}_{hk}$, $k=1,\ldots,K$.

4. Set $\hat{\psi}^{(0)}=\psi^{(0)}$.

5. For iteration $r\geq0$,

a. update the conditional probabilities ($\lambda_{hk}^{(r)}$) for the set of EM households and the importance weights ($\omega_{hk}^{(r)}$) for the set of MCEM households:

$\lambda_{hk}^{\left( r \right)}=\frac{L_{h}(\hat{\psi}^{(r)}|observed data, \boldsymbol{U}_{hk}^{\boldsymbol{*}})}{\sum_{l=1}^{\delta_{h}} L_{h}(\hat{\psi}^{\left( r \right)}|observed data, \boldsymbol{U}_{hl}^{\boldsymbol{*}})}, k=1,\ldots,\delta_{h}$

and

$\omega_{hk}^{\left( r \right)}=\frac{L_{h}(\hat{\psi}^{(r)}|observed data, {\hat{\boldsymbol{U}}}_{hk})}{L_{h}(\hat{\psi}^{\left( 0 \right)}|observed data, {\hat{\boldsymbol{U}}}_{hk})}, k=1,\ldots,K$,

respectively.

b. Maximize

$$Q\left( \psi,\hat{\psi}^{\left( r \right)} \right)=\sum_{h\in{(\delta}_{h}=1)} \ln L_{h}\left( \psi| observed data \right)+$$

$$\sum_{h\in{(1>\delta}_{h}\leq J)} \sum_{k=1}^{\delta_{h}} \lambda_{hk}^{\left( r \right)}\ln L_{h}\left( \psi| observed data,\boldsymbol{U}_{hk}^{\boldsymbol{*}} \right)+$$

$$\sum_{h\in{(\delta}_{h}>J)} \frac{1}{\sum_{k=1}^{K} \omega_{hk}^{(r)}}\sum_{k=1}^{K} \omega_{hk}^{(r)}\ln L_{h}\left( \psi| observed data,{\hat{\boldsymbol{U}}}_{hk} \right)$$

with regard to $\psi$ to find $\hat{\psi}^{(r+1)}$, repeating this step until convergence is achieved for $\psi$.

Due to the relatively small amount of missing information per households, the MCEM step was not required for this analysis.

**2. Estimating the empirical infectious period distribution**

Among the 224 infectious household contacts (*i.e.*, excluding primary and co-primary cases), the onset of shedding was observed in the 138 (62%) for whom at least one stool specimen / rectal swab collected prior to the onset date was negative for *V. cholerae*. Multiple longitudinally-collected stool specimen / rectal swab samples were available for each of these 138 infectious household contacts. Relative to the date of the onset of shedding (day 0 for each individual), we calculate the proportion of specimens collected on each day that were positive for *V. cholerae* (any serogroup-serotype). For this analysis, the probability density function for the infectious period is derived by fitting a Loess kernel smoothing function (bandwidth = 0.4) in Stata v12 (StataCorp, College Station, TX) to the proportion of specimens positive for *V. cholerae* by day since onset of shedding.

**BIBLIOGRAPHIC REFERENCES**

1. Yang Y, Longini IM, Jr., Halloran ME, Obenchain V (2012) A hybrid EM and Monte Carlo EM algorithm and its application to analysis of transmission of infectious diseases. Biometrics 68: 1238-1249.

2. Yang Y, Ira M. Longini J, Halloran ME (2006) Design and evaluation of prophylactic interventions using infectious disease incidence data from close contact groups. Applied Statistics 55: 317-330.

3. Fox JP, Cooney MK, Hall CE, Foy HM (1982) Influenzavirus infections in Seattle families, 1975-1979. II. Pattern of infection in invaded households and relation of age and prior antibody to occurrence of infection and related illness. Am J Epidemiol 116: 228-242.

4. Fox JP, Hall CE, Cooney MK, Foy HM (1982) Influenzavirus infections in Seattle families, 1975-1979. I. Study design, methods and the occurrence of infections by time and age. Am J Epidemiol 116: 212-227.

5. Sack DA, Sack RB, Nair GB, Siddique AK (2004) Cholera. Lancet 363: 223-233.

6. Longini IM, Jr., Nizam A, Ali M, Yunus M, Shenvi N, et al. (2007) Controlling endemic cholera with oral vaccines. PLoS Med 4: e336.

7. Levine RA, Casella G (2001) Implementations of the Monte Carlo EM algorithm. Journal of Computational and Graphical Statistics 10: 422-439.

**Figure S1. Proportion of stool/rectal swab specimens positive for *Vibrio cholerae* (all serogroup-serotypes) by day since onset of shedding (Day 0).** Results were only included for specimens collected from non-primary cases for whom at least one prior specimen was negative for cholera vibrios (N=138). The gray numbers located just above the x-axis denote the number of specimens available for each day since onset of shedding.


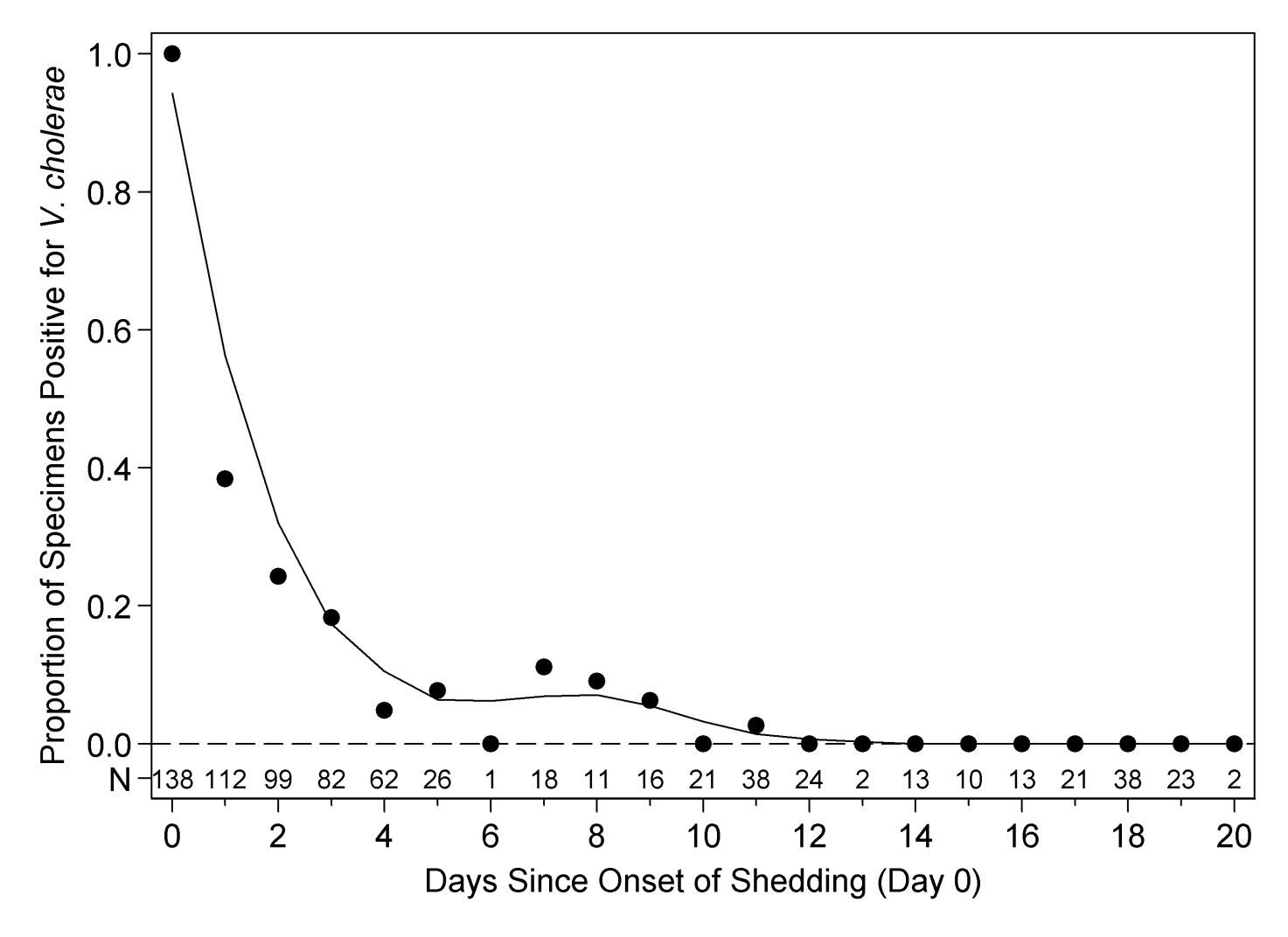


**Figure S2. Community probability of infection (CPI) estimates (squares) and 95% confidence intervals (error bars) for cholera infections (all serogroup-serotypes), by calendar month of exposure.** The horizontal gray line indicates a null CPI value of 0%, corresponding to the situation where no infection of members of study households would be attributed to sources of exposure located outside of the household. Month names (x-axis) are provided in temporal order using standard three-letter abbreviations.


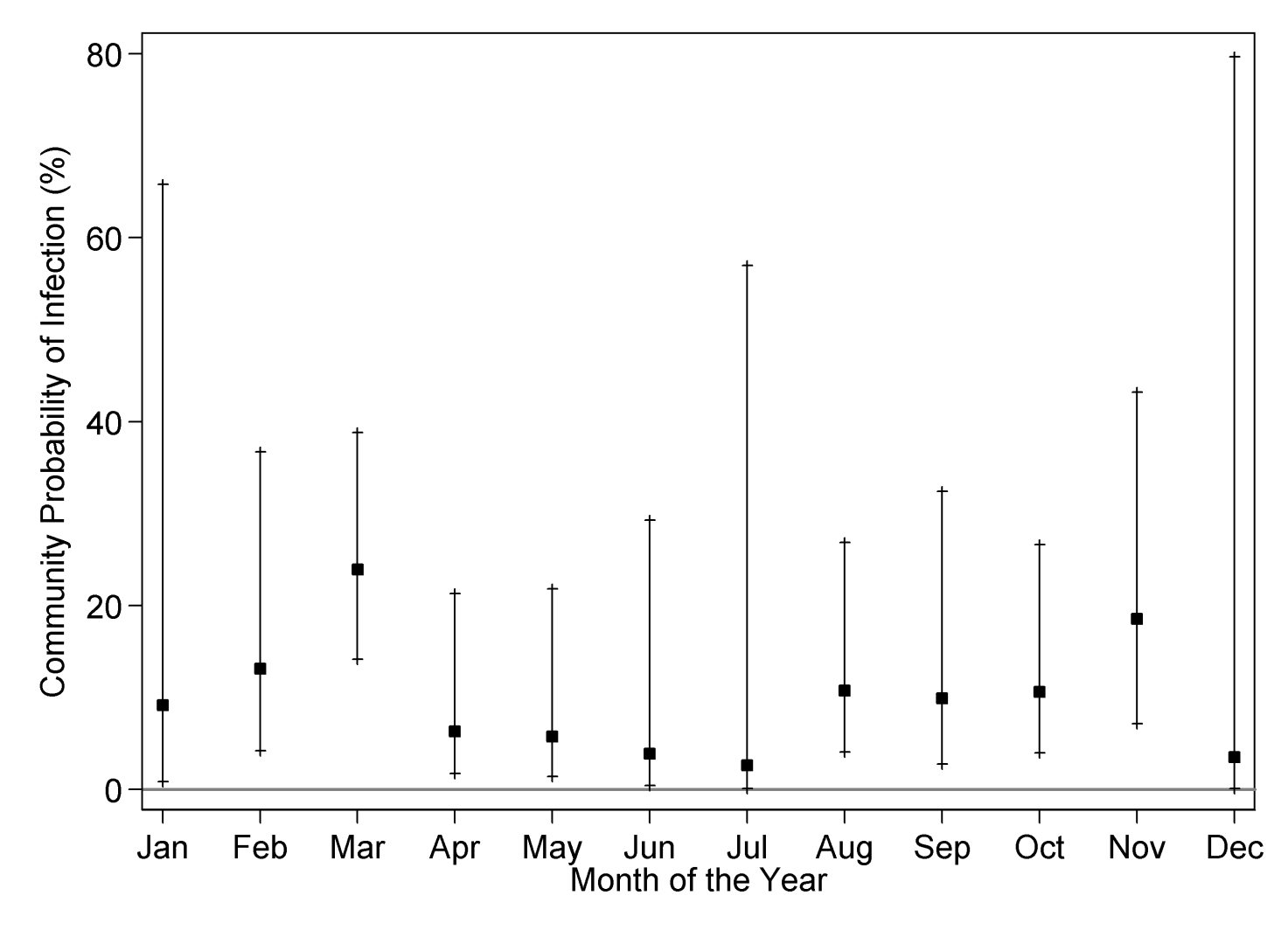


**Figure S3. A comparison of the observed final size frequency distribution for cholera infection (all serogroup-serotypes) among all members of the study households (bar) to the point estimates and 95% confidence intervals (vertical error bars) for the expected distributions under the $\boldsymbol{p}_{\boldsymbol{v}}$-and-$\boldsymbol{b}_{\boldsymbol{v}}$ (cross) and $\boldsymbol{b}_{\boldsymbol{v}}$-only (triangle) models.** Frequency distributions are all scaled to the size of the study population (364 households) and organized by the size of the enrolled household membership and the number of cholera infections that occurred among these individuals by the end of follow-up. Expected distributions are based upon 1500 simulated epidemics in a synthetic population that was identically structured to the study population. Serogroup-serotype specific hazards of infection were used for the simulations, but missing serogroup-serotype information for a proportion of the observed infections (Table S1 in Text S1), necessitated assessment of model fit using the final size distribution for *V. cholerae* infection of any serogroup-serotype.


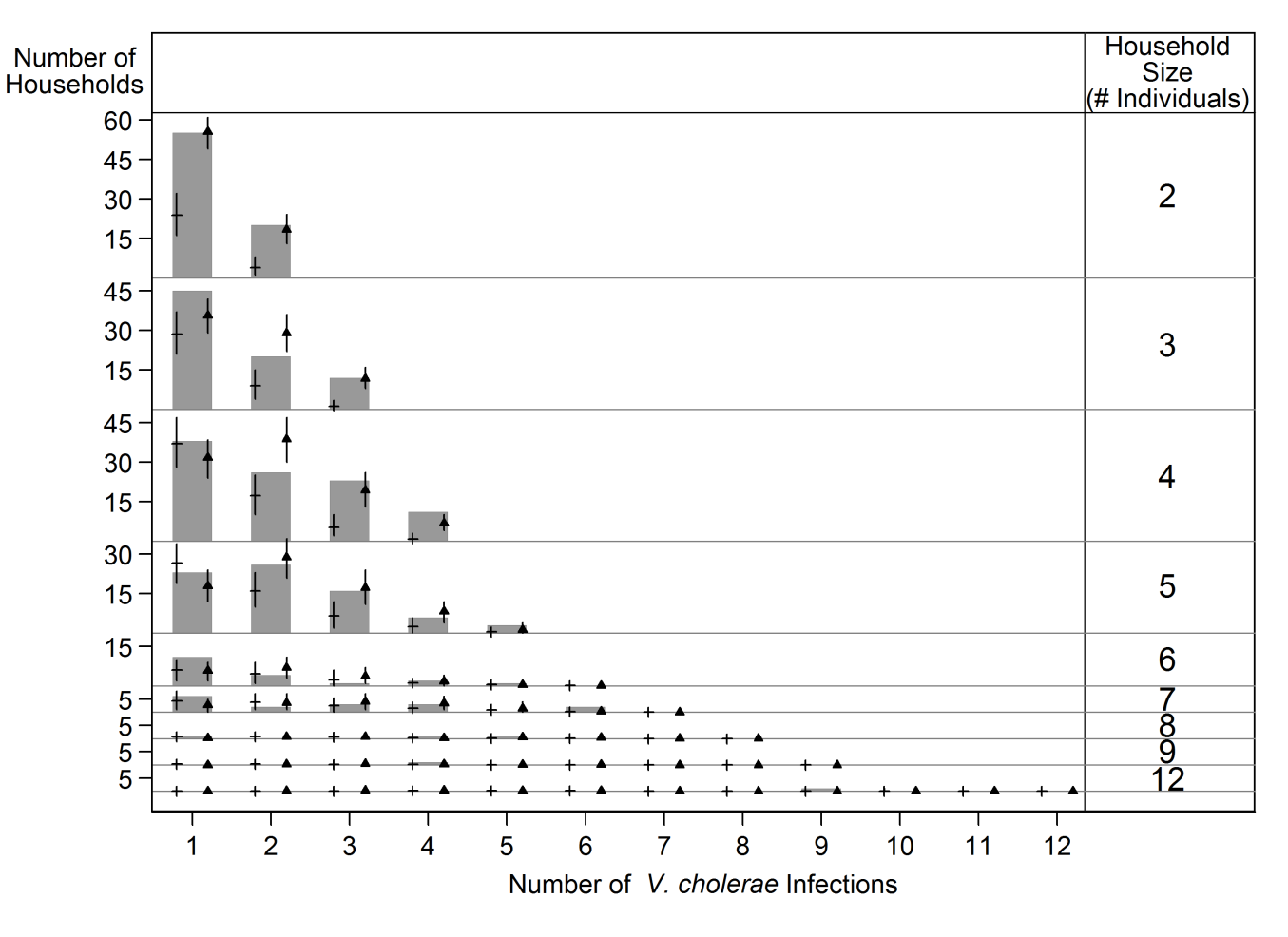


**Table S1. The frequency of study households by the number of household contacts and cholera infections among these contacts, further stratified by the observed serogroup-serotype of infection.**

| **Number of Cholera Infections Among Enrolled Members** | **Observed Serogroup-Serotypes of the Infections among Enrolled Household Members** | | | | | | | | | | | | | | | | | | | | | | | | |
| --- | --- | --- | --- | --- | --- | --- | --- | --- | --- | --- | --- | --- | --- | --- | --- | --- | --- | --- | --- | --- | --- | --- | --- | --- | --- |
|  | **O1 El Tor Ogawa (N=115)** | | | | | | | **O1 El Tor Inaba (N=174)** | | | | | | | **O139 (N=57)** | | | | | | | **Mixed^a^ (N=18)** | | | |
|  | **Number of Enrolled Members**  **(Complete : Missing)^b^** | | | | | | | **Number of Enrolled Members**  **(Complete : Missing)** | | | | | | | **Number of Enrolled Members**  **(Complete : Missing)** | | | | | | | **Number of Enrolled Members**  **(Complete : Missing)** | | | |
|  | **2** | **3** | **4** | **5** | **6** | **7** | **8** | **2** | **3** | **4** | **5** | **6** | **7** | **8** | **2** | **3** | **4** | **5** | **6** | **7** | **12** | **2** | **4** | **5** | **9** |
|  |  |  |  |  |  |  |  |  |  |  |  |  |  |  |  |  |  |  |  |  |  |  |  |  |  |
| **1** | 15:0 | 16:0 | 10:0 | 13:0 | 1:0 | 1:0 | 1:0 | 33:0 | 22:0 | 22:0 | 6:0 | 8:0 | 4:0 |  | 7:0 | 7:0 | 6:0 | 4:0 | 2:0 | 1:0 |  |  |  |  |  |
| **2** | 1:2 | 4:5 | 8:3 | 5:3 | 0:1 | 0:1 |  | 3:0 | 2:5 | 7:3 | 8:7 | 2:1 | 0:1 |  | 6:2 | 2:2 | 1:3 | 1:0 |  |  |  | 6:0 | 1:0 | 2:0 |  |
| **3** |  | 2:1 | 6:3 | 1:3 |  | 1:1 |  |  | 4:4 | 3:6 | 5:4 |  | 1:0 |  |  | 1:0 | 2:0 | 1:0 | 1:0 |  |  |  | 3:0 | 2:0 |  |
| **4** |  |  | 0:2 | 0:1 |  | 1:0 | 1:0 |  |  | 3:2 | 1:3 |  | 0:1 |  |  |  | 2:0 |  | 2:0 | 0:1 |  |  | 0:2 | 1:0 | 0:1 |
| **5** |  |  |  | 1:0 |  |  |  |  |  |  | 0:1 |  |  | 0:1 |  |  |  | 1:0 | 0:1 |  |  |  |  |  |  |
| **6** |  |  |  |  |  | 0:1 |  |  |  |  |  |  | 1:0 |  |  |  |  |  |  |  |  |  |  |  |  |
| **9** |  |  |  |  |  |  |  |  |  |  |  |  |  |  |  |  |  |  |  |  | 1:0 |  |  |  |  |

*Footnotes*

N, Total number of households represented in a serogroup-serotype column.

^a^ Cholera Infections with different serogroup-serotypes observed within the same household.

^b^ Complete=serogroup-serotype was observed for every infection in a household. Missing = serogroup-serotype was unobserved for at least one infection in a household.
